# Vimentin as a Cap of Invisibility: Proposed Role of Vimentin in Rabbit Hemorrhagic Disease Virus (RHDV) Infection

**DOI:** 10.3390/v13071416

**Published:** 2021-07-20

**Authors:** Małgorzata Blatkiewicz, Beata Hukowska-Szematowicz

**Affiliations:** 1 Institute of Biology, University of Szczecin, 71-412 Szczecin, Poland; 2 Molecular Biology and Biotechnology Center, University of Szczecin, 71-412 Szczecin, Poland

**Keywords:** vimentin, rabbit hemorrhagic disease virus (RHDV), rabbit hemorrhagic disease (RHD), gene expression, pathogenesis

## Abstract

Vimentin is an intermediate filament, a cytoskeleton protein expressed mainly in cells of mesenchymal origin. Increasing evidence indicates that vimentin could play a key role in viral infections. Therefore, changes in tissue and extracellular vimentin expression and associated signal trails may determine/protect the fate of cells and the progression of disease caused by viral infection. Rabbit hemorrhagic disease virus (RHDV), genotype GI.1, is an etiological agent that causes a severe and highly lethal disease—RHD (rabbit hemorrhagic disease). This article evaluates the gene and protein expression of vimentin in the tissues (liver, lungs, spleen, and kidneys) and serum of rabbits experimentally infected with two RHDV variants (GI.1a). The VIM mRNA expression levels in the tissues were determined using reverse transcription quantitative real-time PCR (RT-qPCR). In addition, the amount of vimentin protein in the serum was analyzed by an ELISA test. We observed significantly elevated expression levels of VIM mRNA and protein in the liver and kidney tissues of infected rather than healthy rabbits. In addition, VIM mRNA expression was increased in the lung tissues; meanwhile, we observed only protein-enhanced vimentin in the spleen. The obtained results are significant and promising, as they indicate the role of vimentin in RHDV infection and the course of RHD. The role of vimentin in RHDV infection could potentially rely on the one hand, on creating a cap of invisibility against the intracellular viral spread, or, on the other hand, after the damage of cells, vimentin could act as a signal of tissue damage.

## 1. Introduction

Vimentin is a multifunctional protein whose primary function is maintaining cell shape and cytoskeletal stability. The vimentin protein is responsible for epithelial-to-mesenchymal transition (EMT). The 466 amino acid protein is coded by the vimentin gene (*VIM*), mostly expressed in a broad spectrum of cells including lymphocytes, neutrophils, activated macrophages, skeletal muscle cells, and platelets [1,2,3,4,5,6]. The cellular localization of vimentin is directly related to its function. The protein forms networks around the cell nucleus and extends from there to the entire cytoplasm, creating a scaffold for cell organelles [7,8]. *VIM* is also used as a diagnostic biomarker of EMT in oncogenesis and cancer progression [9]. Regarding EMT, cells lose their epithelial characteristics, especially polarity, and obtain a migratory behavior [10]. This leads to them altering their shape and exhibiting increased motility [10]. A growing body of evidence indicates the importance of vimentin overexpression in viral diseases with a rapid onset and long course [11,12,13,14,15,16,17,18,19,20]. Vimentin may also be secreted outside the cell, exposed on the surface of activated macrophages and neutrophils [2,6,21]. Despite the limited evidence, it has been shown that extracellular vimentin has a pleiotropic action [22,23]. It is involved in multiple pathophysiological processes related to immune cell activation, pathogen recognition and interaction, and tissue repair [22,23].

Rabbit hemorrhagic disease virus (RHDV), according to the new classification system, is identified as *Lagovirus europaeus* GI.1/RHDV (rabbit hemorrhagic disease virus 1), a single-stranded, positive-sense RNA virus and an etiological agent that causes a severe and highly lethal disease—RHD [24,25]. Within one to three days after infection via oral or nasal route transmission in animals, the first clinical signs of the disease, such as apathy, fever, and respiratory disorders, appear [26]. The highest viral titers are observed in the liver, spleen, lungs, kidneys, and trachea, which is mostly used for the diagnosis process. Nevertheless, RHDV could also be detected in biological fluids (such as serum) [27,28,29]. RHD was first described in 1984, and for almost 40 years has spread around the world, decimating farms and causing numerous financial losses [26]. Unfortunately, the molecular pathomechanism of RHD is still poorly understood, mainly due to the lack of a robust cell culture system for the propagation of the virus [25,30].

Herein, we broadly investigated the gene and protein expression of vimentin in different types of rabbit tissues and serum during the rapid development of rabbit hemorrhagic disease.

## 2. Materials and Methods

### 2.1. Materials

#### 2.1.1. Animals

This study was performed on male and female healthy, conventional, mixed-breed *Oryctolagus cuniculus* rabbits. The animals were purchased from a licensed breeder and were kept under constant care from veterinary and animal husbandry staff. The animals were not vaccinated against RHDV; nevertheless, before the experiment, all animals were tested for RHDV antibodies using an ELISA test (Institutio Zooprofilattico Sperimentale, Teramo, Italy). The animals all experienced appropriate conditions (humidity, lighting, size of cages, freshwater, and certified food) in accordance with the recommended Polish standards developed in line with the European Union Directive. The experiment was approved by the local ethics committee in Szczecin (no. 1/2009, 26.01.2009). During the experiment, the clinical conditions of the infected and control animals were assessed. Clinical signs in the infected animals and mortality were recorded.

#### 2.1.2. RHDV (Rabbit Hemorrhagic Disease Virus)

In the experiment, two variants of RHDV were used: (I) GI.1a/Erfurt (Germany, 2000) and (II) GI.1a/Rossi (Germany, 2000). The rabbits were divided into three groups: (I) Control animals (*n* = 10); (II) rabbits infected with GI.1a/Erfurt (*n* = 10), called the Erfurt group; (III) rabbits infected with GI.1a/Rossi (*n* = 10), called the Rossi group. The experimental animals were infected by intramuscular injection of the selected virus, while the control group was injected with a form of PBS (phosphate-buffered saline) as a placebo.

#### 2.1.3. Tissue Samples

Tissue samples from the liver, lungs, spleen, and kidneys were obtained from the infected rabbits (*n* = 20) post-mortem and from healthy rabbits (*n* = 10) after euthanasia. Each tissue sample was washed in cold PBS and immediately placed in liquid nitrogen. Frozen tissue samples were stored at −80 °C until RNA or protein extraction.

#### 2.1.4. Serum Samples

Venous blood samples were collected from both infected and healthy rabbits by a cannula from their ear vein into an untreated (anti-coagulant-free) tube. From each infected rabbit, blood was collected before virus administration at 0 h and after virus administration at 8, 12, 24, 36, 48, 52, 56, and 60 h. From healthy rabbits, blood was collected only on the first day of the experiment at 0 h. Serum was freshly isolated from coagulated blood by undisrupted incubation of the samples at room temperature for 20 min. Next, the samples were centrifuged at 2000× *g* for 20 min at 4 °C to separate the serum. The isolated serum was immediately aliquoted and stored at −80 °C until the experiments were performed. 

### 2.2. Methods

#### 2.2.1. RNA Extraction and Quantification of Gene Expression

Total RNA was extracted from the tissues of the infected and healthy rabbits using RNeasy Micro Kit (Qiagen, Hilden, Germany) following the manufacturer’s protocol. cDNA synthesis was carried out with a SuperScript™ VILO™ cDNA Synthesis Kit (Invitrogen, Waltham, MA, USA), according to the manufacturer’s recommendation. These cDNA samples were stored at −20 °C. The expression of *VIM* and the control *18S RNA* transcripts were measured with TaqMan Gene Expression Assays (Applied Biosystems, Waltham, MA, USA) by quantitative real-time PCR (Applied Biosystems, Waltham, MA, USA). The relative expression of the target genes was calculated with the ΔΔCt quantification method.

#### 2.2.2. ELISA Assay

The levels of vimentin protein in the serum, as well as in the liver, lung, spleen, and kidney tissues, were determined by a VIM ELISA test (MyBioSource, San Diego, CA, USA, #MBS1601587). The tissue specimens were prepared following the manufacturer’s protocol. First, the tissues were homogenized on ice with PBS with a glass homogenizer. Next, the samples were incubated on ice for 20 min and then frozen at −20 °C. All samples were then centrifuged at 3000 rpm for 20 min at 4 °C. The supernatant was aliquoted into a fresh, chilled tube and stored at −80 °C until the experiments were performed.

#### 2.2.3. Analysis of VIM Networks Using the STRING Web Portal

Functional protein partners for VIM were identified using the Search Tool for the Retrieval of Interacting Genes (STRING) (version 11.0) analysis web portal (https://string-db.org/; accessed on 24 June 2021) [31]. The score of the minimum required interaction was of medium confidence (0.4).

#### 2.2.4. Statistics

The data were evaluated as a mean ± standard error (SEM) for continuous variables and analyzed using (one-way/two-way) ANOVAs. StatView version 5.0 (SAS Institute, Cary, NC, USA) was used for all analyses. Comparisons between the groups were made using the nonparametric Mann–Whitney test. A *p*-value <0.05 was considered statistically significant.

## 3. Results

### 3.1. Tissue VIM Distribution 

#### 3.1.1. Vimentin Gene and Protein Expression in the Tissues

**Figure 1:**
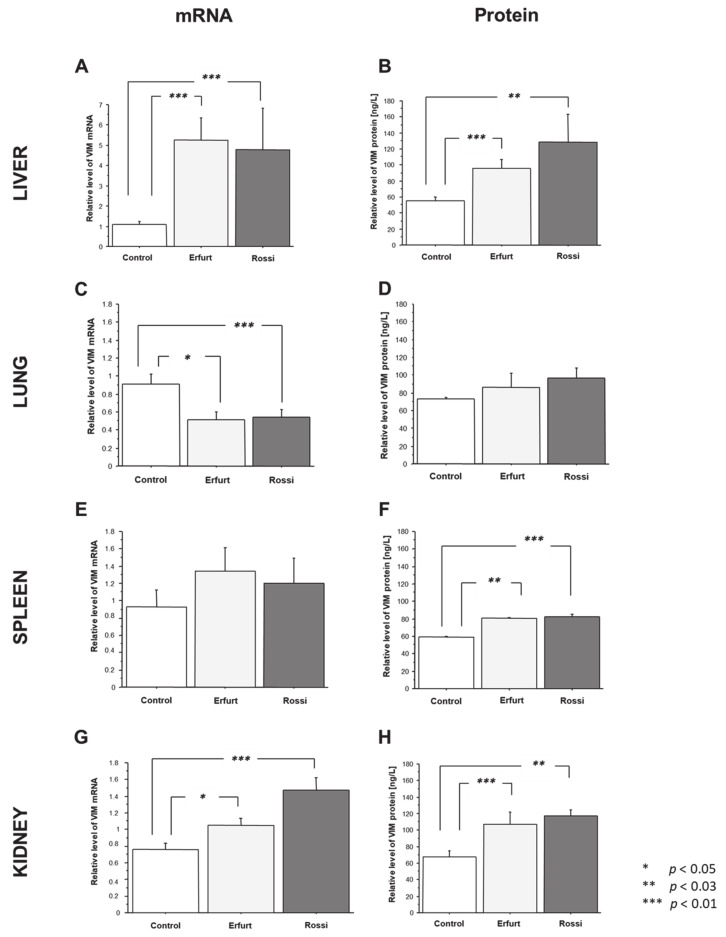
Gene (**A**,**C**,**E**,**G**) and protein (**B**,**D**,**F**,**H**) expression of vimentin in the liver (**A**,**B**), lung (**C**,**D**), spleen (**E**,**F**), and kidney (**G**,**H**) tissues of the rabbits infected with two strains of rabbit hemorrhagic disease virus (RHDV) (Erfurt and Rossi) in comparison to the healthy control. Data are shown as the mean ± standard error, and *p* < 0.05 was considered significant.

To examine the expression patterns of the assessed gene during RHDV infection, we first assessed the *VIM* mRNA levels in the different tissues and compared these findings to the levels obtained in the tissues of the healthy controls. Then, we evaluated the expression of the VIM mRNA product, vimentin protein, in the same way. The mRNA and protein expression of vimentin were analyzed in the liver, lung, spleen, and kidney tissue samples from the RHDV-infected rabbits (two strains—Erfurt and Rossi) and the control tissues (from uninfected, healthy rabbits) (Figure 1).

We observed the highest relative level of VIM mRNA in the liver tissues of the infected animals among all analyzed tissues. The relative gene expression of *VIM* in the liver tissues was significantly increased in both of the infected groups of animals, i.e., Erfurt (a fold-change of 5, *p* = 0.0007) and Rossi (a fold-change of 4.4, *p* = 0.02), compared to the healthy rabbits’ livers (Figure 1A). The protein analysis showed that the vimentin expression in the liver tissues was enhanced in the Erfurt (a fold-change 1.7, *p* = 0.01) and the Rossi (a fold-change of 2.3, *p* = 0.03) groups (Figure 1B).

Downregulation of VIM mRNA during viral infection of RHDVa was detected in the lung tissues (40% reduction for both strains: *p* = 0.04 for Erfurt and *p* = 0.02 for Rossi vs. the control) (Figure 1C). However, the protein level of VIM in the lung tissues was uniformly expressed in both of the infected groups of rabbits (Erfurt *p* = 0.4 and Rossi *p* = 0.1) (Figure 1D).

Compared to the control, the level of *VIM* mRNA was unchanged in the spleen tissues from both of the infected groups of rabbits (Erfurt *p* = 0.2 and Rossi *p* = 0.2) (Figure 1E). Meanwhile, the protein expression of vimentin in the spleen tissues was significantly increased during viral infection (a fold-change of 1.4 (*p* = 0.004) for Erfurt, and a fold-change of 1.4 (*p* = 0.01) for Rossi) (Figure 1F).

The VIM mRNA level was significantly increased in the kidneys of the rabbits infected with the Erfurt and Rossi RHDVa strains, with a 1.4- (*p* = 0.04) and 1.9-fold (*p* = 0.006) increase, respectively (Figure 1G). In agreement with quantitative molecular evaluations, the analyses of protein expression demonstrated a higher level of vimentin protein in the kidney tissue samples during RHDVa infection (a fold-change of 1.8 (*p* = 0.04) for Erfurt, and a fold-change 2 (*p* = 0.01) for Rossi) (Figure 1H).

#### 3.1.2. Vimentin Protein Expression in Serum

**Figure 2:**
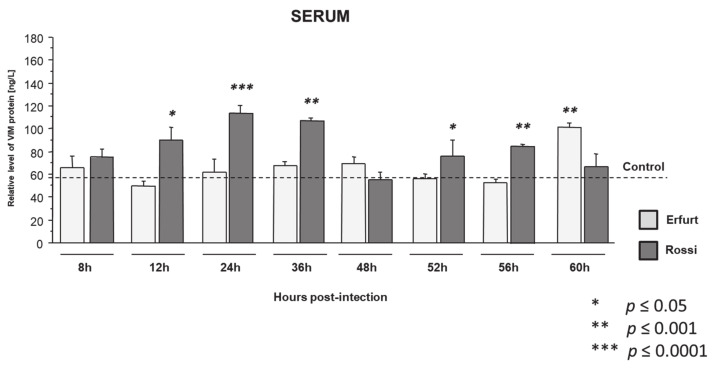
Protein expression of vimentin in the serum during RHDV infection. The boxes indicate the protein level of vimentin of the rabbits infected with the two strains of RHDV (Erfurt and Rossi) in comparison to the healthy controls. Data are shown as the mean ± standard error, and *p* < 0.05 was considered significant.

The levels of vimentin in the serum of the rabbits infected with Erfurt and Rossi RHDV strains over a 60-h time period is shown in Figure 2. Our analysis showed that two strains exhibited a different expression profile of vimentin protein in the serum. The Erfurt strain demonstrated increased vimentin expression only at the end of the rabbits’ lives/experiment (60 h) (*p* = 0.0006). Meanwhile, during the Rossi strain infection, increased vimentin expression was indicated in 12 h (*p* = 0.001), 24 h (*p* = 0.0001), 36 h (*p* = 0.0003), 52 h (*p* = 0.04), and 56 h (*p* = 0.001) post-infection in comparison to the healthy controls. 

#### 3.1.3. STRING Analysis of VIM of *Oryctolagus cuniculus*

**Figure 3:**
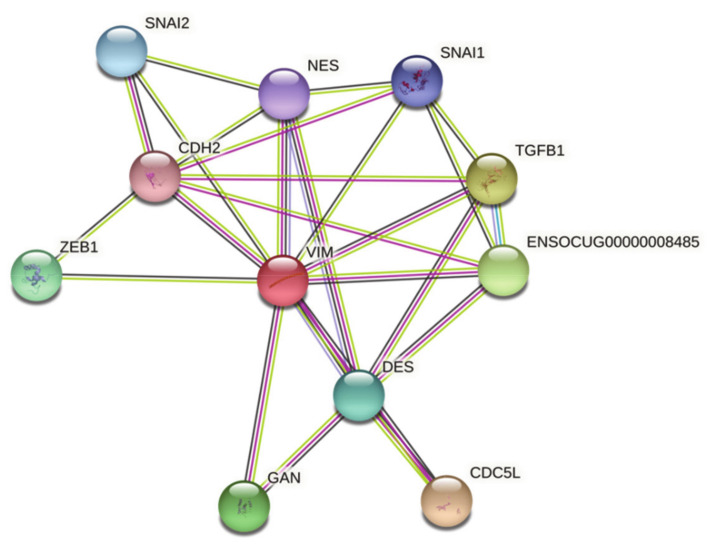
Search Tool for the Retrieval of Interacting Genes (STRING) analysis of *VIM*. Interacting nodes are displayed in colored circles. All abbreviations are listed in the dedicated subsection at the end of the document.

Further analysis, using the STRING database, identified known interactions with *VIM* for 10 discovered genes (Figure 3). *VIM*, as the center node, was associated with transcriptional factors (e.g., SNAI1 and SNAI2), muscle-specific IF desmin (DES), gigaxonin (GAN), nestin (NES), and transforming growth factor beta 1 (TGFB1), being a main anti-inflammatory cytokine.

### 3.2. Clinical Sings of RHDV Infection

During the experiment, the animals showed clinical signs of RHD, including apathy, gelled feces, no response to external stimuli, and rapid breathing. For the Erfurt strain, we indicate 100% mortality between 3- and 4-days post-infection, whereas, for the same time, the mortality of the Rossi strain has 40%.

## 4. Discussion

According to the multifunctional role of vimentin in pathogen infection, we hypothesized that vimentin may play a role in the response to RHDV infection and the course of RHD, which could be a potential target in antiviral strategies.

In this work, we investigated the expression of vimentin at the mRNA and protein levels in the liver, lung, spleen, and kidney tissues and serum of rabbits infected with two different strains of RHDV (Erfurt and Rossi) compared to uninfected, healthy controls. For the first time, we proved that in the tissues of rabbits infected with both the Erfurt and Rossi strains, the *VIM* mRNA expression was elevated in the liver and kidney tissues. In these tissues, mRNA overexpression corresponded with an elevated expression of vimentin protein. Moreover, structural changes in these organs corresponded with high viral titers. The analysis did not reveal any significant differences in the mRNA expression levels of *VIM* in the spleen tissues of infected and uninfected animals. At the same time, the protein expression of vimentin in the spleen was significantly elevated for both analyzed strains. Only in the lung tissues did the expression of *VIM* decrease (for both strains), which translates into the protein level of vimentin. Meanwhile, the serum expression of vimentin depended on the duration of the infection and the strain used in the experiment. Both strains showed different patterns of vimentin expression profile in the serum. For animals infected with the Erfurt strain, characterized by rapid mortality (100%, between 3- and 4-days post-infection), vimentin expression was mainly comparable to the uninfected controls, with elevated protein expression only at 60 h post-infection. Meanwhile, during the Rossi strain infection (40% mortality between 3- and 4-days post-infection), where the animals survived for up to 72 h post-infection, the animals showed an increased expression of vimentin already in the first hours of the experiment (12, 24, and 36 h), with the highest amount of vimentin protein in the serum at 24 h post-infection. 

Based on our previous research [32] of miRNA expression in tissues of rabbits infected with RHDV we firstly hypothesized that expression of vimentin could be correlated with expression of ocu-miR-122-5p, responsible for the regulation of the apoptotic process. In the analysis so far during the RHDV infection, we did not show significant differences in ocu-miR-122-5p expression in liver tissues, impacting VIM expression [32]. Nevertheless, the downregulation of ocu-miR-122-5p may promote cell proliferation and migration by promoting EMT activity. It is known that the extracellular localization of vimentin plays a role as an attachment site for severe viral proteins, promoting cell binding [33]. Furthermore, circulating vimentin may have an origin in apoptotic immune cells (lymphocytes, neutrophils, and macrophages) and hepatocytes, which may have a source in RHDV-induced apoptosis and necrosis [34,35,36,37]. Our analysis showed that overexpression of ocu-miR-122-5p is comparable to overexpression of relisted serum vimentin protein. Therefore, we assume that present in the serum of infected rabbits, extracellular vimentin has originated in apoptotic immune cells and other tissues (especially the liver). Moreover, extracellular vimentin could also act as a signal of tissue damage in the course of RHD, especially in the Rossi strain. However, the role of vimentin depends on the particular viral model and may interact manifold, which needs to be further investigated.

The second hypothesis is correlated with the possible mechanism of vimentin’s role in the immune response and interaction with pathogens, also with RHDV. What is interesting is that the exemplified literature reports proved opposite functions of vimentin, depending on the type of virus. Some viruses can interact with vimentin or modify its cytoskeletal behavior in host cells, disrupting the virus entry, intracellular transportation, maturation, and release [11,38,39,40,41]. Schafer et al. [12] reported that vimentin might interact as an extracellular receptor for viruses. Additionally, the absence of vimentin in knockout mice results in insensitivity to inflammation and acute lung injury (associated with COVID-19) [11,42]. Moreover, drugs that are effective in fighting COVID-19 also reduce vimentin expression. The next theory indicates that perhaps the binding of the virus to the extracellular receptors promotes the signaling tracks, leading to intracellular reorganization and redistribution of the endoplasmic reticulum around the perinuclear region. A consequence of this is anchoring of the virus and forming viral titers, observed in Denga virus (DENV), DNV2 infection, and similarly in Japanese encephalitis virus [13,14,15,16]. Another hypothesis by Schafer et al. [12] suggests that vimentin may play a revised function as a restriction factor, disrupting the interaction between the virus and its receptors. This mechanism has been observed in human papillomavirus (HPV) and leads to limitation of the translocation of the virus into the nucleus. In light of the presented hypotheses, it is hard to state our results in contrast to other evidence because the role of intermediate filaments (such as vimentin) in RHDV infection has not been investigated.

Our last hypothesis is correlated with RNA viruses and their proteins, which could activate key regulatory pathways to promote EMT. It is known that a few non-oncoviruses, such as Ebola virus (EBOV), human cytomegalovirus (HCMV), respiratory syncytial virus (RSV), and human immunodeficiency virus (HIV), induce the EMT process [17,18,19,20]. According to this, changes in the expression of transcriptional factors induce EMT, and the EMT process could also be a potential trick of the cells inhibiting virus entry. Due to this fact, vimentin is a cap of invisibility against intracellular viral spread, and the EMT mechanism may protect the cells from altering the tropism of viruses. Furthermore, decreased expression of transcriptional factor (SNAI1, SNAI2) during pathogen infection may suppress *VIM* expression and reduce pathogen translocation into the cells. We want to believe that the phenomenon known as a cap of invisibility may be a new potential mechanism of cell self-protection system against the RHDV intracellular spread. Also, intracellular vimentin overexpression may implicate virus fusion, replication, and assembly [11].

We are aware that our research has some limitations. The first is the lack of data from the vimentin expression in tissues in several time points post-infection. The second limitation is the shortage of molecular analysis revealing the vimentin localization in cells and tissue during the RHDV infection. Further data collection is required to determine precisely the role of vimentin in the course of RHDV infection, which is included in our further research plans. Unfortunately, we were unable to investigate the relationships between vimentin and their target genes by other molecular methods like immunohistochemistry due to the limitation of financial resources.

Given our findings, the results provide the first step toward enhancing our understanding of vimentin’s mechanism during RHDV infection. In light of presented results and according to the performed STRING analysis, further studies are needed to address our hypothesis and to analyze the genes involved in EMT activation. Moreover, it is necessary to show the localization of vimentin and RHDV molecules in tissues during various infections to confirm or deny the presence of RHDV molecules in the cells after mesenchymal transition and to show host–pathogen interactions. 

## 5. Conclusions

Our research focused on an extensive analysis of the vimentin gene and protein expression in various types of rabbit tissues and serum during the rapid development of RHDV infection. Herein, we proved that RHDV infection indirectly affects intermediate filament expression, even if not remodeling the filaments themselves. The role of vimentin in RHDV infection could rely, on the one hand, on creating a cap of invisibility against intracellular viral spread. On the other hand, after the damage of cells, vimentin could act as a factor for tissue damage. This proposal indicates the pluripotential hallmark of vimentin during viral infection, which needs to be investigated in the future.

## Data Availability

All data are available after personal email request from the authors Małgorzata Blatkiewicz (blatkiewicz.malgorzata@gmail.com) and Beata Hukowska-Szematowicz (beata.hukowska-szematowicz@usz.edu.pl).

## Abbreviations

EMT—Epithelial-to-mesenchymal transition
ENSOCUG00000008485—Protein
CDH2—Cadherin 2
CDC5L—Cell division cycle 5-like
COVID-19—Coronavirus disease 2019
DES—Desmin
DENV—Denga virus
EBOV—Ebola virus
GAN—Gigaxonin
HCMV—Human cytomegalovirus
HPV—Human papillomavirus
NES—Nestin
TGFB1—Transforming growth factor beta 1
SNAI1—Snail family zinc finger 1
SNAI2—Snail family zinc finger 2
PBS—Phosphate-buffered saline
RHD—Rabbit hemorrhagic disease
RHDV—Rabbit hemorrhagic disease virus
RSV—Respiratory virus
VIM—Vimentin
ZEB1—Zinc finger E-box binding homeobox 1
